# Modeling and prediction for diesel performance based on deep neural network combined with virtual sample

**DOI:** 10.1038/s41598-021-96259-x

**Published:** 2021-08-18

**Authors:** Hainan Zheng, Honggen Zhou, Chao Kang, Zan Liu, Zhenhuan Dou, Jinfeng Liu, Bingqiang Li, Yu Chen

**Affiliations:** 1 School of Mechanical Engineering, Jiangsu University of Science and Technology, Zhenjiang, China; 2 Shaanxi Diesel Heavy Industry, LTD, Xi’an, China

**Keywords:** Engineering, Mechanical engineering

## Abstract

The performance models are the critical step for condition monitoring and fault diagnosis of diesel engines, and are an important bridge to describe the link between input parameters and targets. Large-scale experimental methods with higher economic costs are often adopted to construct accurate performance models. To ensure the accuracy of the model and reduce the cost of the test, a novel method for modeling the performances of marine diesel engine is proposed based on deep neural network method coupled with virtual sample generation technology. Firstly, according to the practical experience, the four parameters including speed, power, lubricating oil temperature and pressure are selected as the input factors for establishing the performance models. Besides, brake specific fuel consumption, vibration and noise are adopted to assess the status of marine diesel engine. Secondly, small sample experiments for diesel engine are performed under multiple working conditions. Moreover, the experimental sample data are diffused for obtaining valid extended data based on virtual sample generation technology. Then, the performance models are established using the deep neural network method, in which the diffusion data set is adopted to reduce the cost of testing. Finally, the accuracy of the developed model is verified through experiment, and the parametric effects on performances are discussed. The results indicate that the overall prediction accuracy is more than 93%. Moreover, power is the key factor affecting brake specific fuel consumption with a weighting of 30% of the four input factors. While speed is the key factor affecting vibration and noise with a weighting of 30% and 30.5%, respectively.

## Introduction

Diesel engines have been widely used in ships and automobiles due to their large power range, high thermal efficiency, and low fuel consumption rate^1–3^. The status of the diesel engine is intrinsically linked to the controlled parameters during the running process of the diesel engine. To understand the relationships between diesel engine performance and control parameters, many studies have focused on modeling methods based on large sample experiments, theory, and simulation. Recently developments have attracted more scholars to concentrate their studies on this field^4–6^.

Nowadays, numerical simulation method has been applied to deal with complex nonlinear problems of diesel engine. As for the performance prediction of diesel engine, Nahim et al.^7^ presented a numerical simulation model of a marine diesel engine based on physical, semi-physical, mathematical and thermodynamic equations. This model can accurately predict the pressure, temperature, and emissions of diesel engines. Gosala et al.^8^ used a phase-angle approach to build a vibration prediction model. It is demonstrated that the weighted phase-angle approach can accurately and quickly predict the frequencies and relative amplitudes of the vibration. The proposed model can be further used for online, real-time selection of ignition modes under steady-state operating conditions. In terms of parametric optimization of diesel engine, Muse et al.^9^ simplified the in-cylinder combustion process of a diesel engine and developed a 1-dimensional large two-stroke low-speed diesel engine model. Diesel engine characteristics were analyzed by changing the model injection parameters, and optimization of injection characteristics and exhaust timing was achieved, resulting in a 9% reduction in NO_X_ emissions. Besides, based on the GT-SUITE, the simulation models of diesel engines were also established to predict and optimize fuel consumption, noise and emissions^10,11^. The optimization process was carried out through numerical simulation with suitable objective functions aimed at minimizing BSFC while not exceeding the target NO_x_ emission levels. Recently, the numerical simulation method has also been used for fault diagnosis of diesel engine. Rubio et al.^12^ built a one-dimensional thermodynamic model using AVLBoost software. This model was able to effectively simulate 15 typical thermodynamic faults such as turbine failure, exhaust manifold leakage and intake valve seat failure, which provided information for the development of a diesel engine failure simulator. Overall, the numerical simulation approach reduces the amount of expensive experimental trials required to evaluate the performance of the engines. However, as diesel engine systems become increasingly complex, the time for model calculation and optimization increases. This approach becomes gradually unsuitable for large marine diesel engine modeling.

With the development of algorithm theory, intelligent algorithms have been applied to diesel engine performance studies. Aghbashlo et al.^13^ designed a novel method to estimate the exergetic performance of a DI diesel engine based on extreme learning machine with a wavelet transform algorithm. The method allows estimating, optimizing, and controlling the operating performance of a diesel engine in real time under known engine operating conditions and fuel characteristics. Similarly, an adaptive neuro-fuzzy inference system tuned by particle swarm algorithm was used to predict diesel engine performance and emissions by Atarod et al.^14^. The effective prediction of CO_2_, CO, and NO_x_ emissions was achieved after the continuation of the modeling system based on experimental data, which provides a reference for the next optimization of diesel engines. The intelligent algorithm could predict the diesel engine operating parameters very well. Moreover, it also provides ideas and directions to optimize the diesel engine operating parameters and fuel composition^15^. Shin et al.^16^ combined the deep neural network and Bayesian method to optimizing the diesel engine parameters and predicting the NOx transient emission, which enhanced the model stability and accuracy significantly. Dinesha et al.^17^ incorporated cerium oxide (CeO_2_) nanoparticles into diesel for effectively reducing diesel emissions and improving diesel engine performance. Besides, intelligent algorithms are also applied for the fault diagnosis. Zhang et al.^18^ proposed a convolutional neural network-based (CNN) method for diesel engine misfire fault diagnosis. The results showed that the CNN method can accurately detect a complete misfire in one or two cylinders when the diesel engine is operating under steady-state conditions.

From the various literature, it can be observed that many modeling approaches were proposed to discuss diesel engine performance prediction and fault diagnosis. Much research had focused on software simulations or intelligent algorithms based on large samples. However, models obtained by simulation methods often lack the necessary experimental information, resulting in models that fail to fully reflect the effects introduced by interfering factors. Besides, extensive experimental information is necessary to obtained accurate performance models using intelligent algorithms. Therefore, it is necessary to propose a modeling approach that includes a small amount of experimental information to construct an accurate model.

In this paper, a novel method is proposed to establish the performance models of marine diesel engines based on the Deep Neural Network (DNN) method coupled with Virtual Sample Generation (VSG). First, the predicted performances are analyzed, and an experiment is designed. Then, the diffusion trend of experimental data is mined and analyzed based on VSG technology. Finally, the marine diesel engine performance model is established by deep neural network for guaranteeing the accuracy of prediction. Here, the innovation points of this paper are summarized in Table 1 below. In addition, in order to better understand the article, Table 2 of abbreviations and symbol nations of terms has been added.

**Table 1:** Innovation points.

No.	Innovation points
1	In order to reducing the cost of experimental modeling, the DNN coupled with VSG technology is proposed to establish the accurate performance models of the diesel. The models achieved in this paper could reflect the relationship of parameters using small experiment samples
2	The influence law of diesel engine parameters on the performance is obtained based on the proposed models. Moreover, the parametric sensitivity is determined based on impact factors analysis. These would provide a guideline for engineer to optimize and assess performance in practice

**Table 2:** Abbreviation and symbol notations.

**Abbreviation**	
DNN	Deep neural network
VSG	Virtual sample generation
TSA	Trend similarities among attributes
IQR	Interquartile range
MIV	Mean influence value
BSFC	Brake specific fuel consumption (g/kWh)
**Symbol notations**	
*a*_*w*_	Weighted acceleration
*T*	Measurement time
*a*_*tota*l_	Total vibration acceleration
*L*,*U*	Lower limit and upper limit
*MF*	Sample distribution value
*g*(*i*)_*p, q*_	Sample trend value
*S*_*p,q*_	Strength of correlation
*v*	Evaluation symbol
*θ*_*p, q*_	Offset
${v}_{Xp}^{-}$,${v}_{Xp}^{+}$	Possible virtual value
*Ri*_*max,*_* Ri*_*min*_	Predicted results after expanding samples

## Experimental set-up and design

### Experimental equipment

In this paper, the experimental tests are conducted using a 20-cylinder, four-stroke, water-cooled diesel engine, which is provided by Shaanxi Diesel Heavy Industry, LTD. The detailed parameters of experimental equipment are shown in Table 3. During the experimental process, the hydro dynamometer is mounted on the diesel engine to provide different load conditions. Meanwhile, the diesel engine speed is adjusted to achieve different test conditions by controlling the throttle position lever.

**Table 3:** Marine diesel engine specifications.

Parameter	Value
Engine type	4 cycle-20 cylinder
Bore and stroke	230 mm × 230 mm
Type of ignition	Compression ignition
Total working volume	191.2 L
Compression ratio	12:1
Rated power	3750 kW
Rated speed	1455 rpm
Cooling system	Water cooling

### Experimental design

It is well known that the performances of diesel engines are sensitive to power and speed, which could be used for reflecting the operating conditions^19^. Moreover, the oil pressure and temperature are also important to analyze the combustion of the diesel engine^20^. Therefore, power, speed, oil temperature, and pressure are selected as input factors to study parametric effects on the diesel engine in this paper. Meanwhile, three critical parameters including brake specific fuel consumption, vibration, and noise are adopted as the outputs. In the design of experiment, eleven working conditions based on the propulsion characteristic line of the diesel engine are designed to completely investigate the status of the diesel engine during the entire operation. According to the practice experience, the diesel engine speed varies from 600 to 1500 rpm, the lubricating oil temperature range is 77–95 °C, the power range covers 260–4380 kW and lubricating oil temperature range covers 0.3–0.7 MPa. The designed tests are shown in Table 4.

**Table 4:** Experimental design.

No.	Speed (rpm)	Power (kW)	Lubricating oil temperature (°C)	Lubricating oil pressure (MPa)
1	600	260	77	0.41
2	750	500	79	0.48
3	920	940	84	0.56
4	1150	1875	87	0.55
5	1320	2810	88	0.70
6	1455	3750	92	0.69
7	1455	3750	92	0.69
8	1455	3750	92	0.68
9	1455	3750	92	0.67
10	1455	3990	92	0.66
11	1500	4380	94	0.65

### Experimental measurement

**Figure 1:**
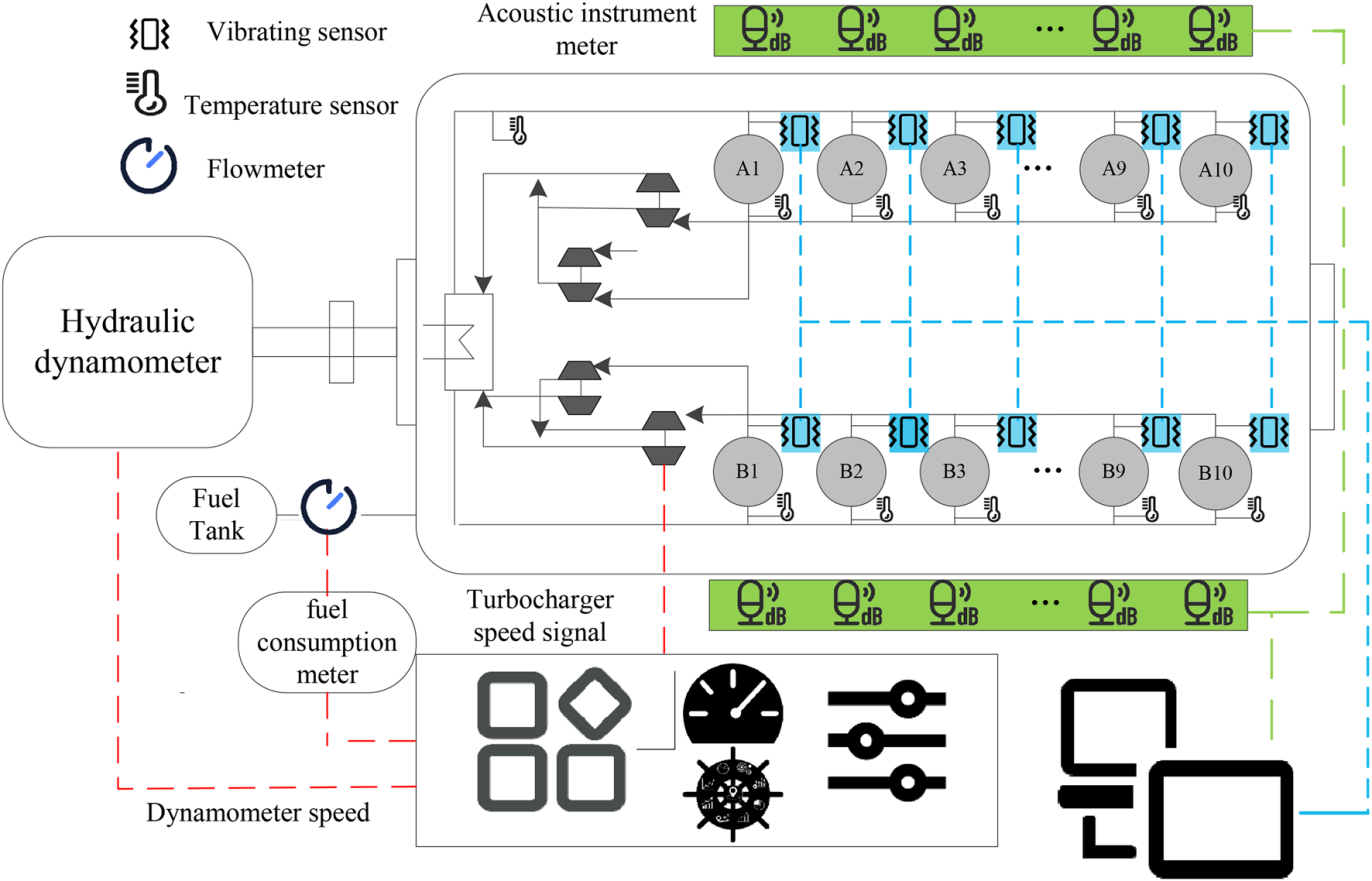
The detailed schematic of the experimental set up.

**Figure 2:**
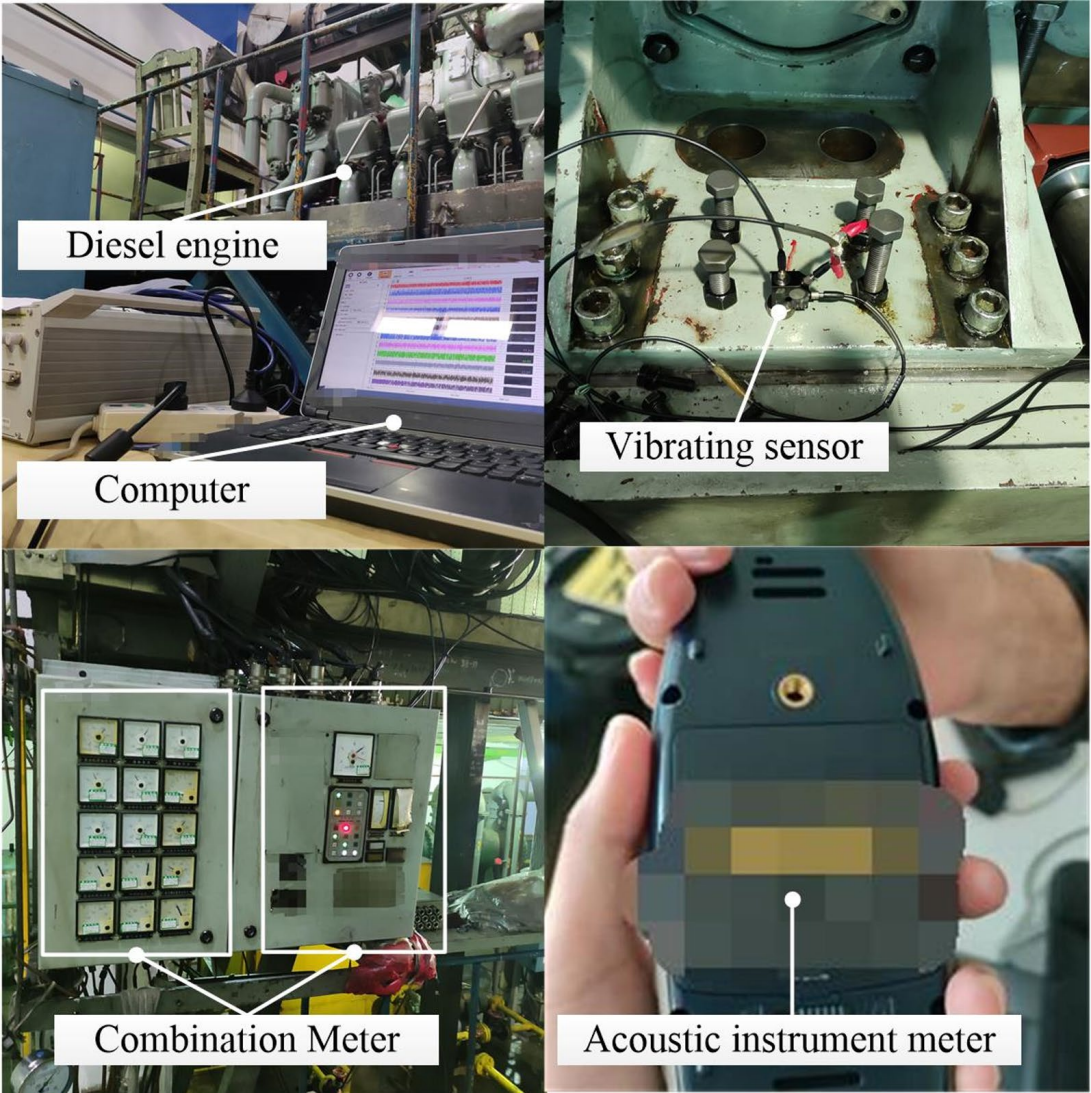
The performances measurement for the multicylinder engine in experiment.

As shown in Fig. 1, the 1A307E vibration sensor is attached to the cylinder head of the diesel engine for measuring the vibration signal. The noise signal is obtained by a BSWA 308 sound level meter at 1 m from the cylinder head. The fuel consumption rate is collected by the fuel consumption meter. During the experiment, the speed and load are controlled to achieve the expected working conditions. Then the speed, power, lubricating oil temperature and pressure are measured by the speed sensor, hydraulic dynamometer, temperature sensor and pressure sensor, respectively. The experimental details of the diesel engine are shown in Fig. 2.

**Figure 3:**
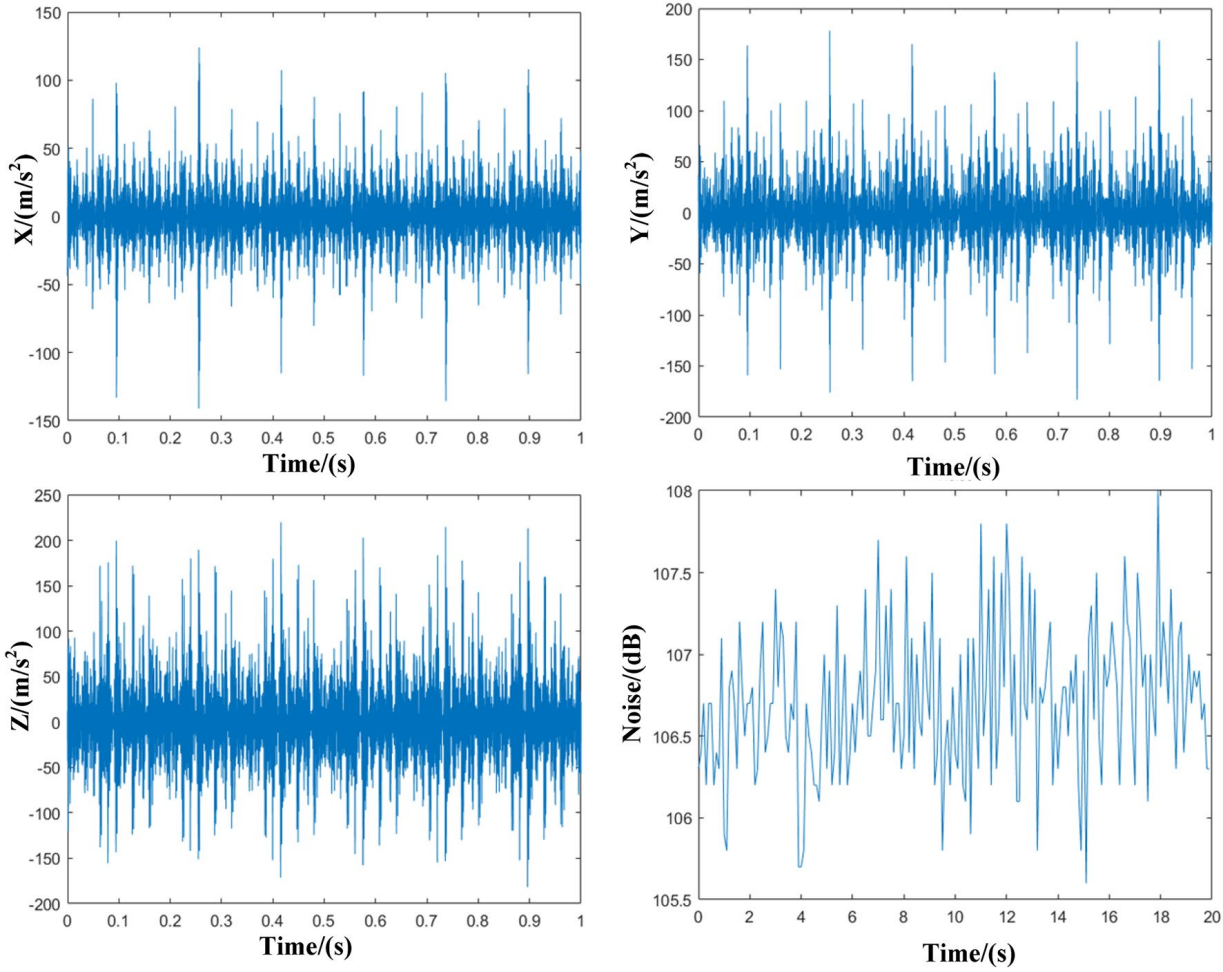
Measurement results of vibration and noise.

Figure 3 shows the noise and vibration results measured in the experiment process. These results are continuous and vary too fast so that it is hard to express the continuous values through a certain model. Thus, the root mean square value is proposed to characterize the state of the diesel engine. The root mean square can be calculated as follows^21^,

1 $$ a_{w} = \sqrt {\frac{1}{T}\int_{0}^{T} {a_{w}^{2} \left( t \right)dt} } , $$

where *a*_*w*_ represents the weighted acceleration and *T* represents measurement time.

Due to the presence of vibration in three directions, the total vibration acceleration is used to evaluate the vibration state of the diesel engine. The acceleration equation is presented in Eq. (2),

2 $$ a_{total} = \sqrt {a_{x}^{2} + a_{y}^{2} + a_{z}^{2} } , $$

where total vibration acceleration (*a*_*tota*l_) is the value to show the combined acceleration of vertical (*a*_*x*_), lateral (*a*_*y*_) and longitudinal (*a*_*z*_).

### Uncertainty analysis

In laboratory experiments, uncertainty analysis deals with evaluating the uncertainty in any measurements. It allows the estimation of the numerical value of a physical variable and how it is affected by errors due to instrumentation. In the present work, the uncertainty of a dependent variable is calculated using errors involved in measuring independent parameters such as power, speed, and lubricating oil temperature. The uncertainty value can be derived by Eq. (3)^17,22^.

3 $$ W_{R} = \left( {\left[ {\frac{\partial R}{{\partial x_{1} }}w_{1} } \right]^{2} + \left[ {\frac{\partial R}{{\partial x_{2} }}w_{2} } \right]^{2} + \cdots + \left[ {\frac{\partial R}{{\partial x_{1} }}w_{n} } \right]^{2} } \right)^{1/2} . $$

The uncertainty values for various parameters are listed in Table 5

**Table 5:** Uncertainty analysis of the measured parameters.

Measured quantity	Percentage uncertainty
Speed	0.64
Power	1.3
Lubricating oil temperature	0.11
Lubricating oil pressure	0.36
BSFC	0.21
Vibration	0.37
Noise	0.17

## Modeling method

**Figure 4:**
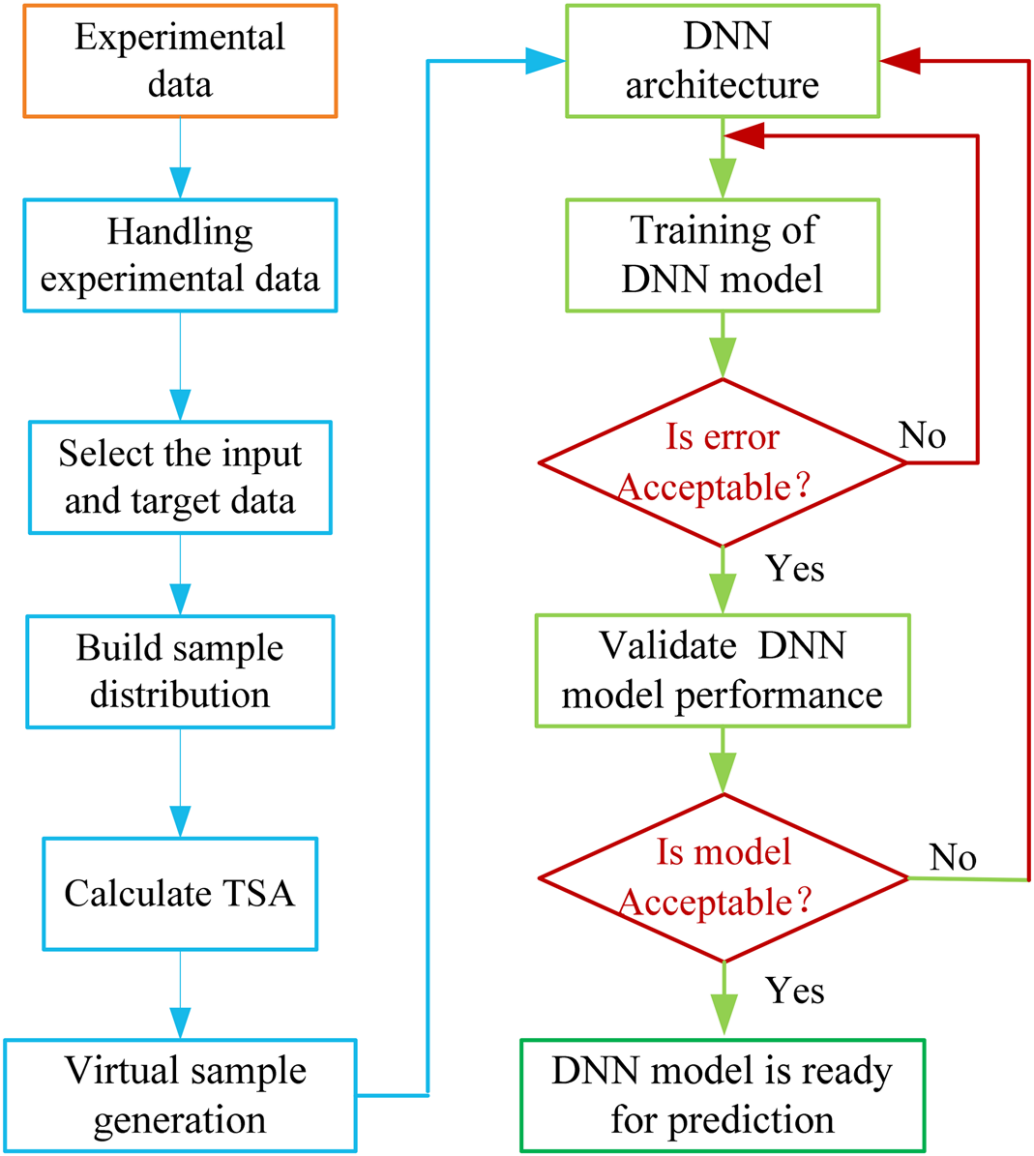
The flowchart of DNN method coupled with VSG.

Compared with other algorithms, the DNN method has a strong advantage for modeling nonlinear complex systems. However, the DNN algorithm requires a large amount of sample data to ensure accuracy of model^23^. To reduce the cost of the diesel experiment, virtual sample generation technology is adopted to diffuse the experimental samples. The virtual sample generation technique is also a current sample diffusion method with high accuracy, and has been used in conjunction with various intelligent algorithms with good results. To clearly illustrate the proposed approach, the flowchart of DNN coupled with VSG is displayed in Fig. 4. Firstly, the target data is achieved through conducting the experiments. Then the sample distribution is determined by dealing with experimental samples. By comparing the magnitude of the values, calculate the trend similarities among attributes (TSA), and the virtual sample data is generated further. Finally, the performance model is obtained by training and validating the sample based on DNN method.

### Virtual sample generation

#### Build sample distribution

In order to achieve the virtual sample, the sample distribution should be firstly determined according to the experimental data. The procedure is given as follows,

Step 1: The test data are collected and divided into input parameters and output parameters of the diesel engine. As for the diesel engine system, the input parameters include speed, power, lubricating oil temperature and pressure. The output parameters are brake specific fuel consumption, total vibration acceleration and noise.

Step 2: Based on the small data sets obtained from the above experiments, seven sample domain boundaries are estimated. For obtaining the boundaries, the range should be achieved firstly. Here the interquartile range (*IQR*) is used to describe the range, which can be derived as follows,

4 $$ IQR = Q_{3} - Q_{1} , $$

where *Q*_1_ is the first quartile of each sample set, and *Q*_3_ is the third quartile of each sample set.

Step 3: The lower limit (*L*) and upper limit (*U*) of the sample domain boundary should be obtained. The reasonable bounds [*L, U*] can be calculated by the following Eqs. (5) and (6),

5 $$ L = \left\{ \begin{gathered} Q_{1} - 1.5 \times IQR,\quad L \le \min \hfill \\ \min ,\quad \, Q_{1} - 1.5 \times IQR > \min , \hfill \\ \end{gathered} \right. $$

6 $$ U = \left\{ \begin{gathered} Q_{3} + 1.5 \times IQR,\quad {\text{U}} \le \max \hfill \\ \max ,\quad \, Q_{3} + 1.5 \times IQR < \max , \hfill \\ \end{gathered} \right. $$

where min and max are the minimum and maximum values of the observations, respectively.

**Figure 5:**
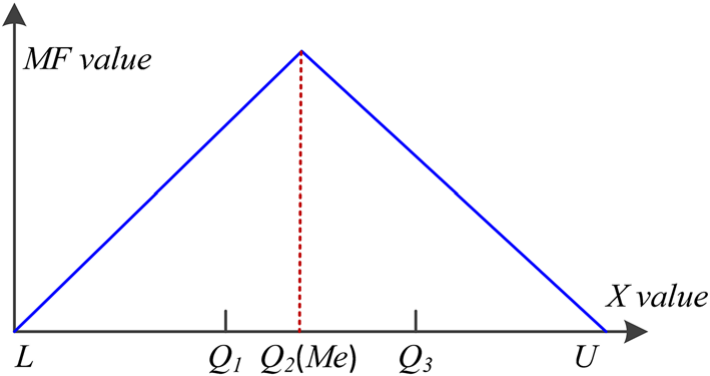
The shapes of attribute sample distribution.

Step 4: Determine the sample distribution *MF*. When the domain bounds of observations are determined, a triangular *MF* based on *L*, *Q*_2_ (*Me*, taken as the location center of sample range as depicted in Fig. 5), and *U* could represent the estimated sample distribution. *MF* is formulated as follows^24,25^,

7 $$ MF\left( x \right) = \left\{ \begin{gathered} \left( {x - L} \right)/\left( {Me - L} \right),\quad L \le x < Me \, \hfill \\ \left( {U - x} \right)/\left( {U - Me} \right),\quad Me \le x < L \, \hfill \\ 0,\quad {\text{otherwise}}. \hfill \\ \end{gathered} \right. $$

Based on the above formula and the measured small data set, the shape of the parameter distribution is plotted and shown in Fig. 5.

#### Calculate trend similarities among attributes

In this section, the trend similarities between attributes are measured according to a non-parametric process^24,25^. First, two parameters *Xp* and *Xq* are selected in two related attribute domains. Here *Xp* is the rotational speed, and *Xq* is an arbitrary parameter. Then the trend assessment function *g*(*i*)_*p*,*q*_ of the *i*-th observation between *X*_*p*_ and *X*_*q*_ can be formulated as,

8 $$ g\left( i \right)_{p,q} = \left\{ \begin{gathered} 1,\quad if\left( {x_{p,i} - Me_{{X_{P} }} } \right)\left( {x_{q,i} - Me_{{X_{q} }} } \right) > 0 \hfill \\ 0,\quad if\left( {x_{p,i} - Me_{{X_{P} }} } \right)\left( {x_{q,i} - Me_{{X_{q} }} } \right) = 0 \hfill \\ - 1,\quad if\left( {x_{p,i} - Me_{{X_{P} }} } \right)\left( {x_{q,i} - Me_{{X_{q} }} } \right) < 0. \hfill \\ \end{gathered} \right. $$

The strength of the trend similarity between *X*_*p*_ and *X*_*q*_ is derived as the average of all available observations, as shown in Eq. (9).

9 $$ S_{p,q} = \frac{1}{n}\sum\limits_{i = 1}^{n} {g\left( i \right)}_{p,q} ,\quad p,q \in \, \left\{ {1,2, \ldots ,m} \right\},p \ne q. $$

Based on the experimental data, the values of *|S*_*p,q*_*|* are obtained as shown in Table 6. Here, the larger this value is, the stronger the correlation between *Xp* and *Xq*.

**Table 6:** The relation strengths of trend similarities among attributes.

Relation strength	Absolute value of *S*(*|S|*)
Low	[0,0.3)
Medium	[0.3,0.7)
High	[0.7,1]

#### Virtual sample generation

Based on the value *g*(*i*)_*p,q*_ obtained above, a value interval is projected to produce a suitable virtual value. The specific steps are given as follows^24,25^:

Step 1: Determine the value of *v*_*Xp*_, and then produce *v*_*Xq*_. Randomly select one temporary value (*tv*) from *U* (*L*_*Xp*_,* U*_*Xp*_), and then calculate the *MF*_*Xp*_(*tv*). Choose a random seed (*rs*) from *U*(0,1) to assess whether *tv* can be kept as a suitable virtual value *v*_*Xp*_.

Step 2: The cumulative distribution function value *F*(*rs*) of the uniform distribution represents the cumulative probability of *rs*. The probability that *rs* is lower than *MF*_*Xp*_(*tv*) is the possibility of the value of *MF*_*Xp*_(*tv*) itself occurring. When *rs* is lower than *MF*_*Xp*_(*tv*), *tv* will thus be kept as *v*_*Xp*_, otherwise, *tv* will be discarded. Therefore, if *MF*_*Xp*_(*tv*) is larger, *tv* will have a higher probability of being *v*_*Xp*_. The evaluation criteria are Eq. (10):

10 $$ v = tv,\quad if\,rs \le MF_{{X_{p} }} \left( {tv} \right). $$

Select the *v*_*Xp*_ value that meets the criteria, and further calculate the offset as given in Eq. (11)

11 $$ \theta_{p,q} = - 0.8 \times \left| {S_{p,q} } \right| + 0.9. $$

The interval bounds $\left[{v}_{Xp}^{-},{v}_{Xp}^{+}\right]$ are given in Eqs. (12) and (13)

12 $$ v_{{X_{p} }}^{ - } = \left\{ \begin{gathered} v_{{X_{p} }} - \theta_{p,q} \left( {U_{{X_{p} }} - L_{{X_{p} }} } \right),\quad v_{{X_{p} }}^{ - } \ge L_{{X_{p} }} \, \hfill \\ L_{{X_{p} }} ,\quad v_{{X_{p} }} - \theta_{p,q} \left( {U_{{X_{p} }} - L_{{X_{p} }} } \right) < L_{{X_{p} }} , \hfill \\ \end{gathered} \right. $$

13 $$ v_{{X_{p} }}^{ + } = \left\{ \begin{gathered} v_{{X_{p} }} + \theta_{p,q} \left( {U_{{X_{p} }} - L_{{X_{p} }} } \right),\quad v_{{X_{p} }}^{ + } \le U_{{X_{p} }} \, \hfill \\ U_{{X_{p} }} ,\quad \, v_{{X_{p} }} + \theta_{p,q} \left( {U_{{X_{p} }} - L_{{X_{p} }} } \right) > U_{{X_{p} }} . \hfill \\ \end{gathered} \right. $$

Then substitute the value ${v}_{Xp}^{-}$ and ${v}_{Xp}^{+}$ into the following equation. The calculation of *MF*(*x*) is given in Eq. (14):

14 $$ v_{{X_{q} }} = \left\{ \begin{gathered} L_{{X_{q} }} + MF\left( {v_{{X_{P} }} } \right)\left( {Me_{{X_{q} }} - L_{{X_{q} }} } \right),\quad L_{{X_{q} }} \le v_{{X_{q} }} \le Me_{{X_{q} }} \hfill \\ U_{{X_{q} }} - MF\left( {v_{{X_{P} }} } \right)\left( {U_{{X_{q} }} - Me_{{X_{q} }} } \right),\quad Me_{{X_{q} }} \le v_{{X_{q} }} \le U_{{X_{q} }} \hfill \\ 0,\quad \, {\text{otherwise}}. \hfill \\ \end{gathered} \right. $$

**Figure 6:**
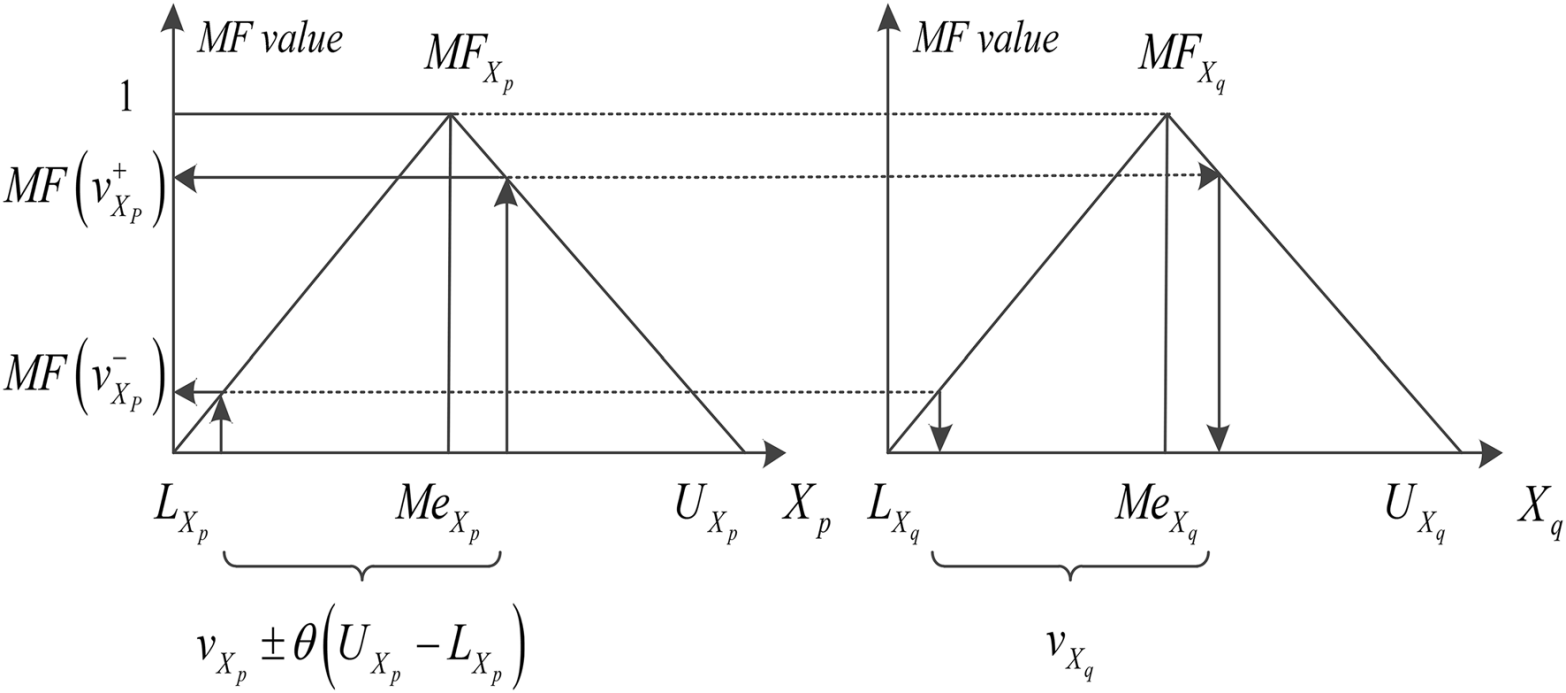
Estimating the possible attribute ranges based on TSA.

Step 3: Sample generates. Based on the above formula, the interval $\left[{v}_{Xp}^{-},{v}_{Xp}^{+}\right]$ is determined, and the random value *v*_*Xq*_ from the interval is selected. The schematic diagram is shown in Fig. 6. According to the above method, all dummy samples are generated in turn.

### Deep neural network model

The deep neural network model is widely used to predict various application results with high accuracy. The basic structure of the deep neural network model comprises one input layer, one output layer, and more than two hidden layers. The DNN algorithm trains internal parameters such as weights and matrix biases according to relationships between input features and output results. The training procedure results in a higher accuracy comparing with the equation-based modeling^26,27^. In this paper, the deep neural network prediction model is built by the following steps.

#### Step 1: Input/output parameters

The input parameters of the network are the operating parameters of the diesel engine, which includes diesel engine speed, power, lubricating oil temperature and pressure. The output parameters of the network are brake specific fuel consumption, vibration, and noise in this paper.

#### Step 2: Normalization process

Since the neural network is a parallel processing system, the network weights are parallel in order of magnitude during its training and prediction. If the difference in the order of magnitude of the input/output parameters of the network is too large, the influence of a smaller order of magnitude parameter on the network weight may be masked by a larger order of magnitude parameter. This would cause a degradation of the network prediction performance. Therefore, it is necessary to normalize the input/output parameters before training the network. The selected normalization function is given as,

15 $$ \overline{x} = - 1 + \frac{{2\left( {x - x_{\min } } \right)}}{{x_{\max } - x_{\min } }}, $$

where *x* is the vector to be normalized, *x*_*max*_ is the maximum value of the sample, *x*_*min*_ is the minimum value of the sample, and $\overline{x}$ is the normalized vector.

#### Step 3: Network structure and network training

Compared with traditional feed-forward neural networks, DNNs have multiple implicit layer structures. Each hidden layer requires the input vector of the previous layer, and performs a nonlinear transformation using the activation function of the hidden layer. Then, the obtained vectors are passed from inputs to the next layer of neurons. Finally, the output is passed to the network through the iterate method. To determine the best network structure, the predicted performance under different numbers of neurons in the hidden layer is first compared. Then, the best network structure is selected for further optimization. In this paper, the training and testing are set to 3:1. The hidden layer is determined to be 4 layers, the number of nodes is 10, and the weight matrix *W* is expressed as,

16 $$ W = \left[ {W^{1} \, W^{2} \, W^{3} \, W^{4} \, W^{5} } \right]{.} $$

The connection weight matrix *W*^*1*^ between the input layer and the hidden layer neurons, and the connection weight matrix *W*^*5*^ between hidden layer and output layer neurons are as follows,

17 $$ W^{1} = \left[ \begin{gathered} W_{1,1}^{1} \, W_{1,2}^{1} \, W_{1,3}^{1} \, W_{1,4}^{1} \hfill \\ W_{2,1}^{1} \, W_{2,2}^{1} \, W_{2,3}^{1} \, W_{2,4}^{1} \hfill \\ \vdots \, \vdots \, \vdots \, \vdots \hfill \\ W_{10,1}^{1} \, W_{10,2}^{1} \, W_{10,3}^{1} \, W_{10,4}^{1} \hfill \\ \end{gathered} \right] $$

18 $$ W^{5} = \left[ \begin{gathered} W_{1,1}^{5} \, W_{1,2}^{5} \, \cdots \, W_{1,10}^{5} \hfill \\ W_{2,1}^{5} \, W_{2,2}^{5} \, \cdots \, W_{2,10}^{5} \hfill \\ W_{3,1}^{5} \, W_{3,2}^{5} \, \cdots \, W_{3,10}^{5} \hfill \\ \end{gathered} \right]. $$

## Result and discussion

### Model accuracy analysis

#### Correlation analysis

**Figure 7:**
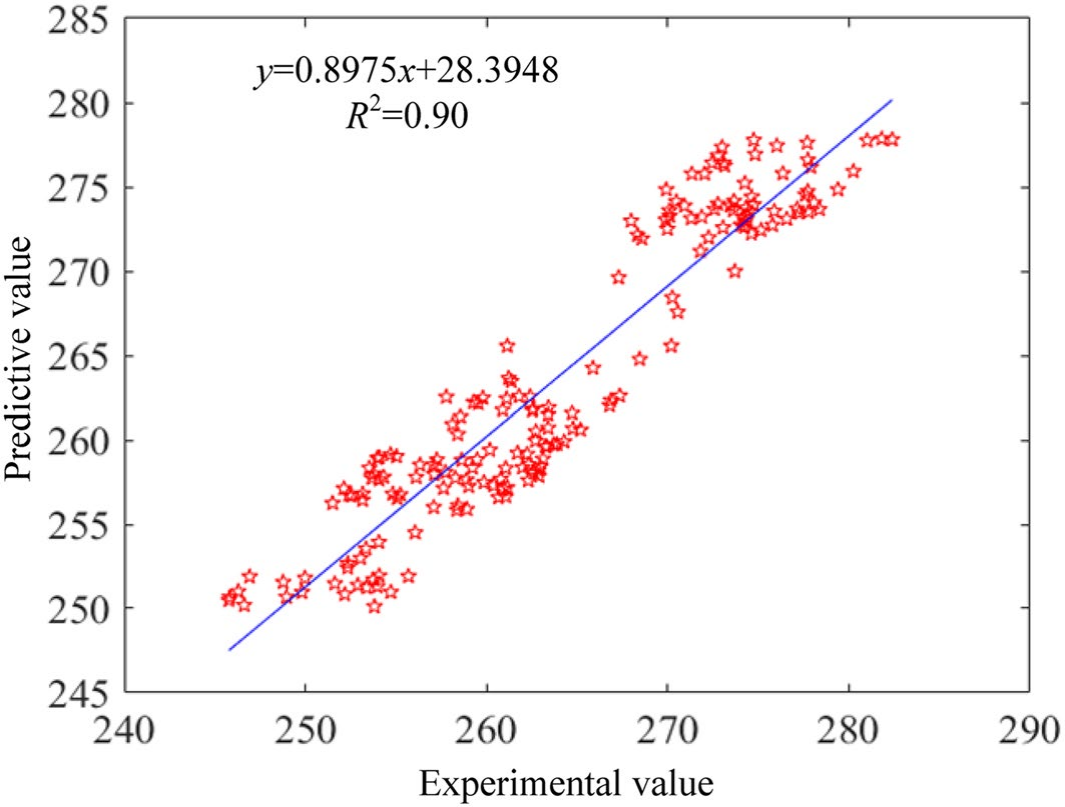
Residual comparison of brake specific fuel consumption between predictive and experimental values.

**Figure 8:**
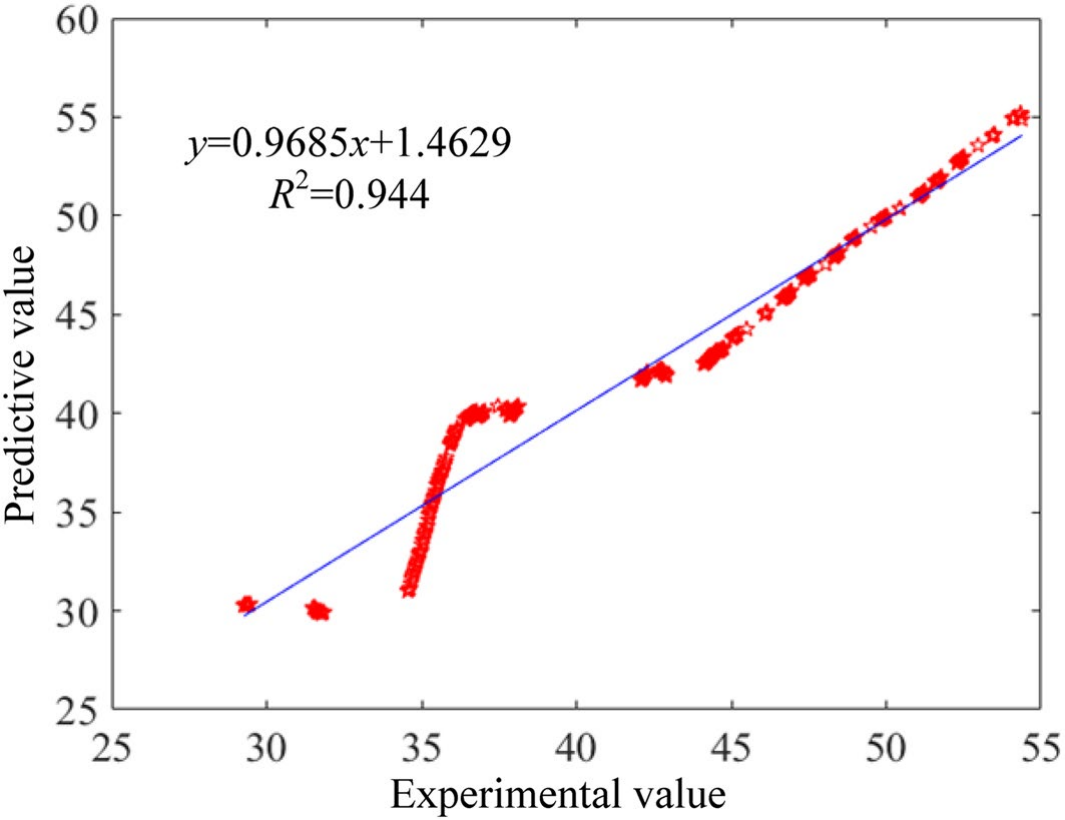
Residual comparison of vibration between predictive and experimental values.

**Figure 9:**
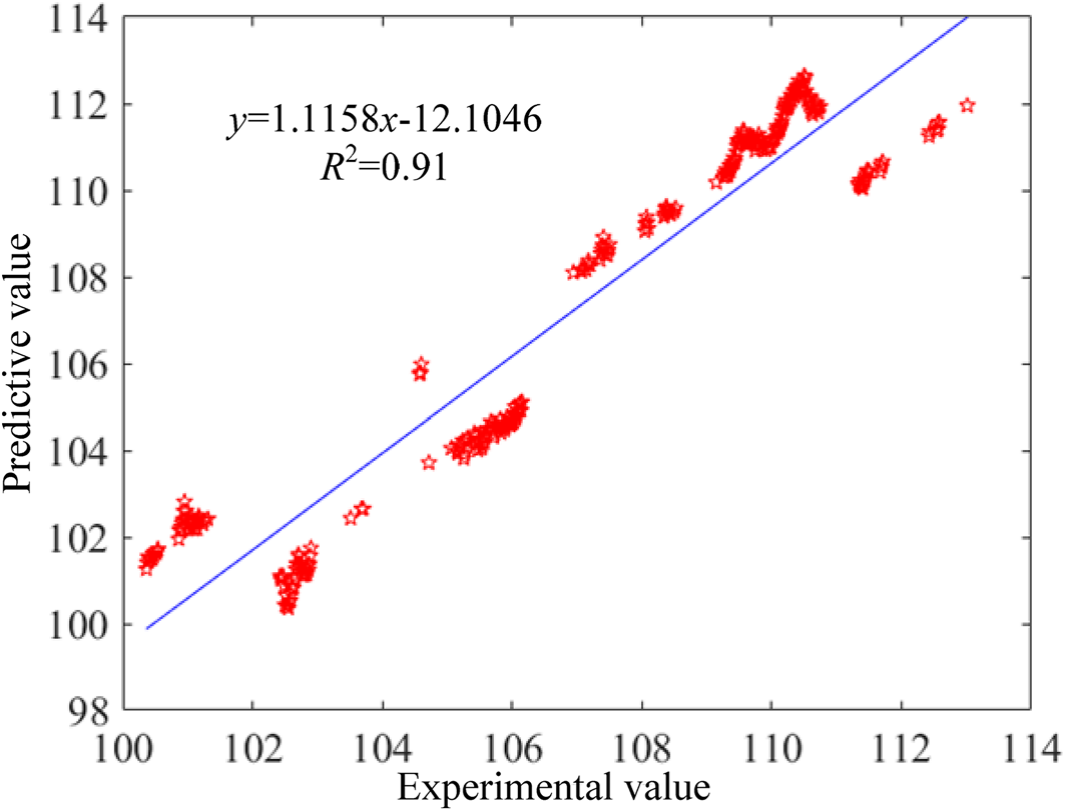
Residual comparison of noise between predictive and experimental values.

Based on the large amount of data generated by VSG, the deep neural network model was trained and tested for prediction accuracy. The residual plots of the brake specific fuel consumption, vibration and noise prediction models are shown from Figs. 7, 8, 9. From the plots, it can be found that the coefficients of determination (R^2^) between the predicted and sample values of fuel consumption rate, vibration and noise are 0.90, 0.94 and 0.91, respectively. That indicates a high correlation between predicted model and experiment data. Besides, the observed and predicted values of the three responses are concentrated around the zero-error line, which fully indicates that the DNN model has a good correlation.

#### Experimental verification

**Figure 10:**
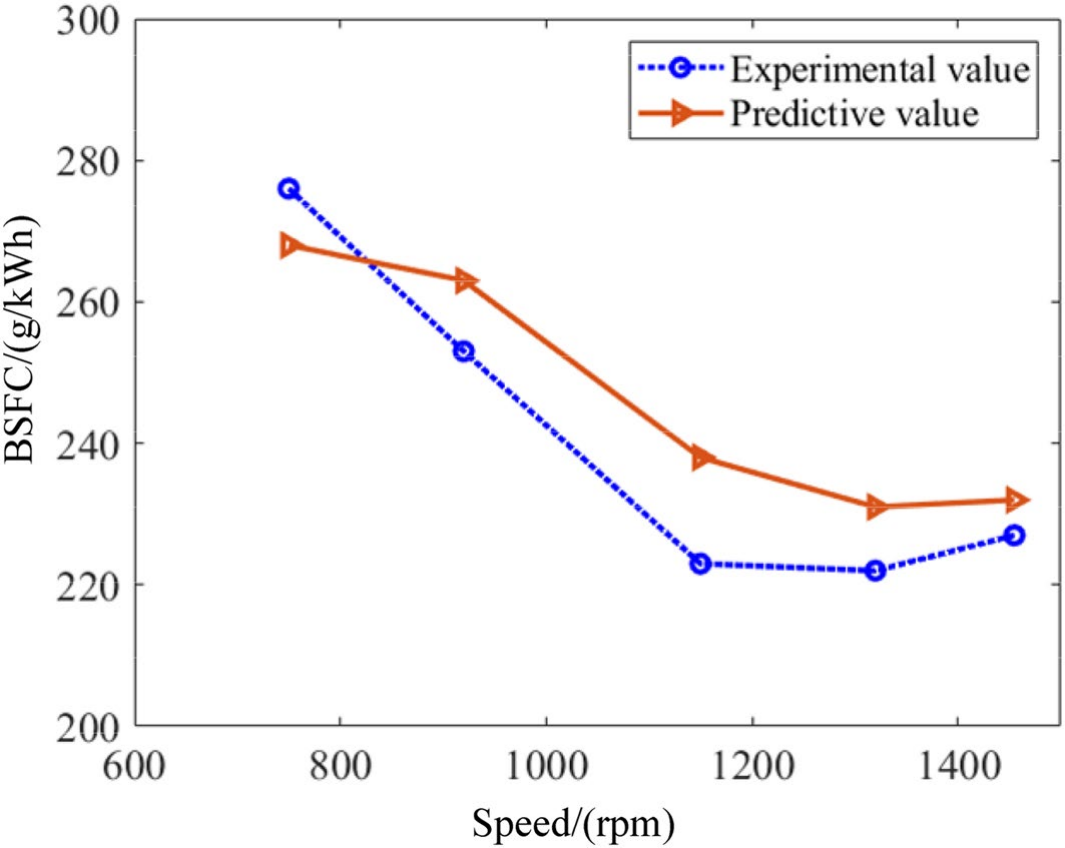
Comparison of brake specific fuel consumption between predictive and experimental values.

**Figure 11:**
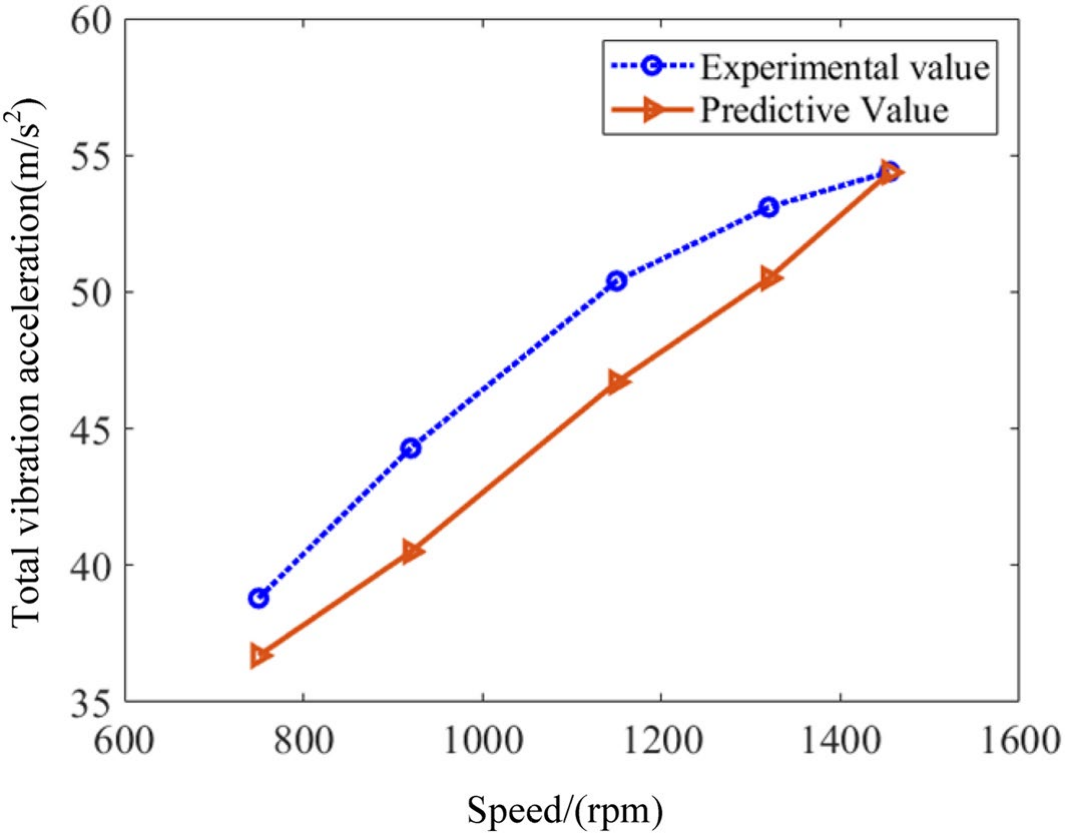
Comparison of vibration between predictive and experimental values.

**Figure 12:**
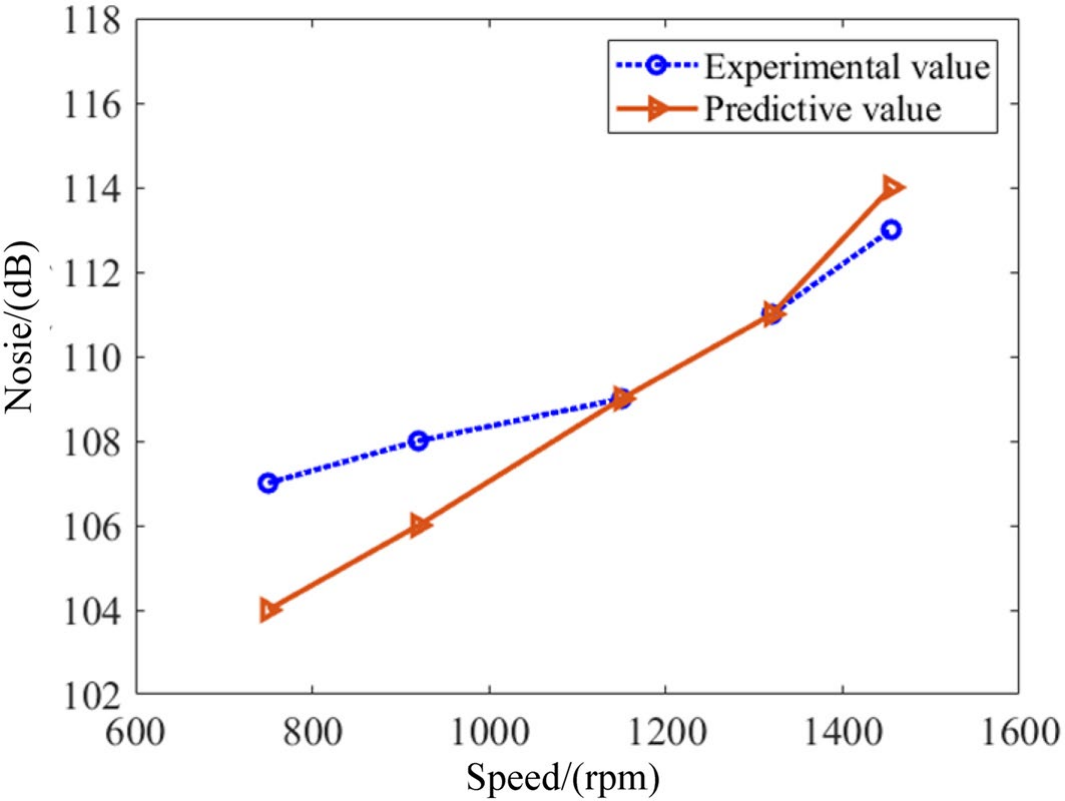
Comparison of noise between predictive and experimental values.

In order to further experimentally verify the reliability of the performance model proposed in this paper, the brake specific fuel consumption, total vibration acceleration, and noise are measured under five working conditions including the speed at 750 rpm, 920 rpm, 1150 rpm, 1320 rpm and 1455 rpm. Figure 10 describes the comparison of the brake specific fuel consumption between experimental and predictive values. As can been seen, the maximum error occurs at 750 rpm, and the error between the predicted and experimental values is close to 7%. When the rotational speed rises to 1300 rpm, the error between the predicted and experimental values is close to 3.9%. Moreover, the trend of experimental and predicted values is generally consistent. Figure 11 depicts the results of vibration. It is found that the trends of the experimental and predicted values of vibration are similar. When the speed increases to 1150 rpm, the error between the predicted and experimental values of the total vibration acceleration is the largest, close to 7%. While the speed reaches 1450 rpm, the error is the smallest, close to 1%. The noise of marine diesel is demonstrated as shown in Fig. 12. It is observed that the predicted results roughly overlap with the experimental values. It means the prediction model is accurate. The maximum error occurs at a speed of 750 rpm, which is close to 2%. The minimum error occurs at a speed of 1150 rpm, which is close to 1%. This result shows the model is very accurate and fully compliant with engineering requirements.

### Effect of input parameters

For the marine diesel engines, the main reason of vibration and noise are piston movement and parts wear. Rotational speed and power are closely related to piston motion. Higher speed and power lead to faster piston motion and increased inertia forces such as crankshaft speed. This leads to high fuel consumption, vibration and noise. As for the oil pressure and temperature, they are related to the wear of parts. When the lubricating oil pressure and temperature are too low, the lubricating oil will not flow smoothly and the diesel engine will not work properly. High oil pressure and temperature will make the oil pump parts overload, the friction surface is not easy to form oil film, resulting in unreliable lubrication, increase parts wear, oil consumption increases. This causes an increase of vibration, noise, and fuel consumption. Therefore, in order to reveal the influence of diesel engine operating parameters on its performance, the diesel engine mechanism is discussed below in conjunction with the prediction model.

#### Brake specific fuel consumption

**Figure 13:**
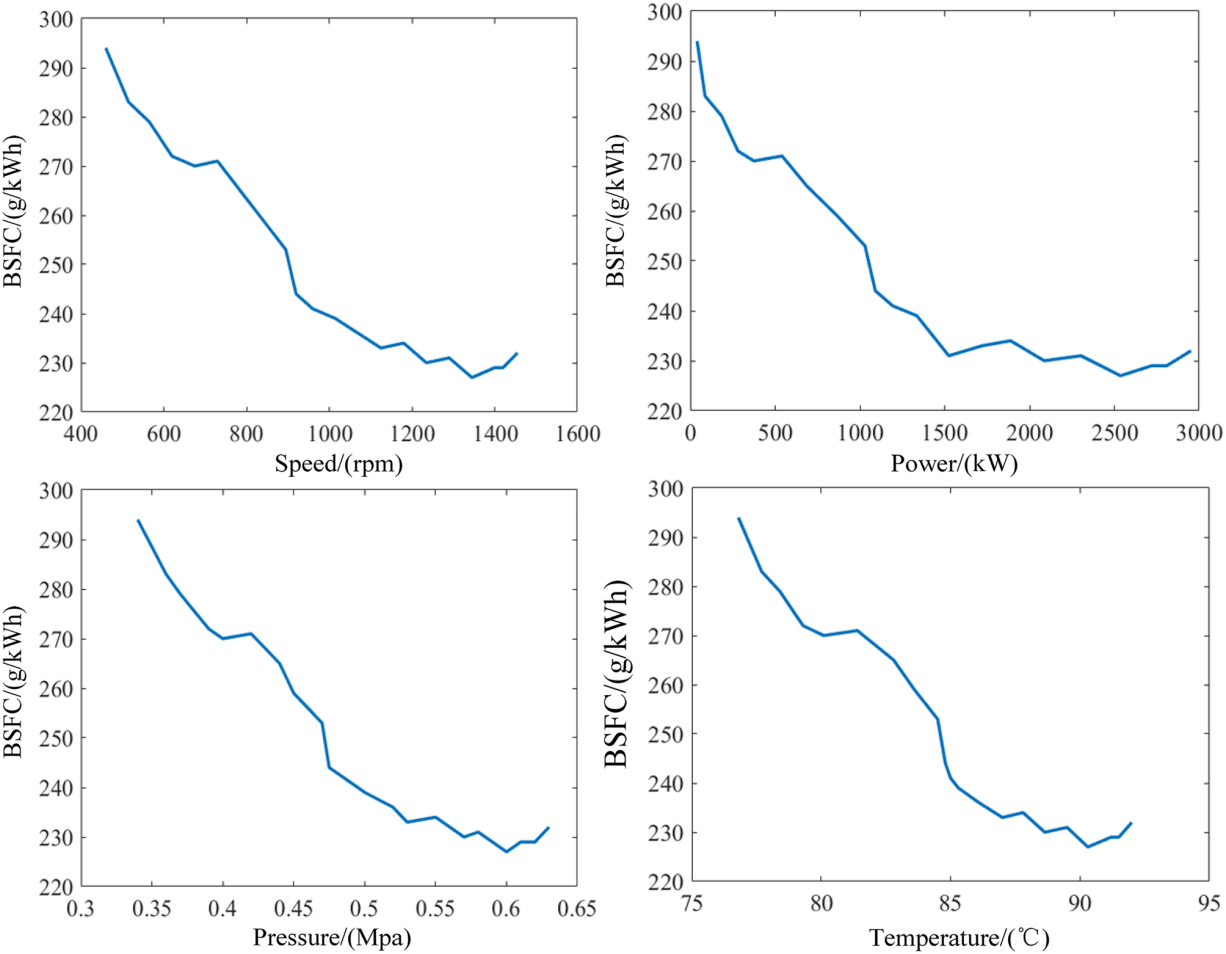
Influence of input parameters on brake specific fuel consumption.

Brake specific fuel consumption is one of the important economic index of diesel engines. In order to improve the power and economy of diesel engines, the effect of parameters on brake specific fuel consumption needs to be studied. Figure 13 shows the effect of input parameters on brake specific fuel consumption. It is found that the brake specific fuel consumption decreases moderately for all parameters as the input parameters increase. That is due to the losses of the low mechanical efficiency and increased leakage at the start of the diesel engine, which results in higher brake specific fuel consumption values. The trend in brake specific fuel consumption is generally consistent with that in the previous literature^28^. However, the brake specific fuel consumption drops slowly after 1000 rpm. It can be explained that the brake specific fuel consumption is relatively low as the diesel engine gradually reach near rated operating conditions. When the diesel engine speed reaches 1350 rpm, fuel consumption is minimal. Subsequently, the fuel consumption increases and shows an upward trend.

#### Vibration of the engine

**Figure 14:**
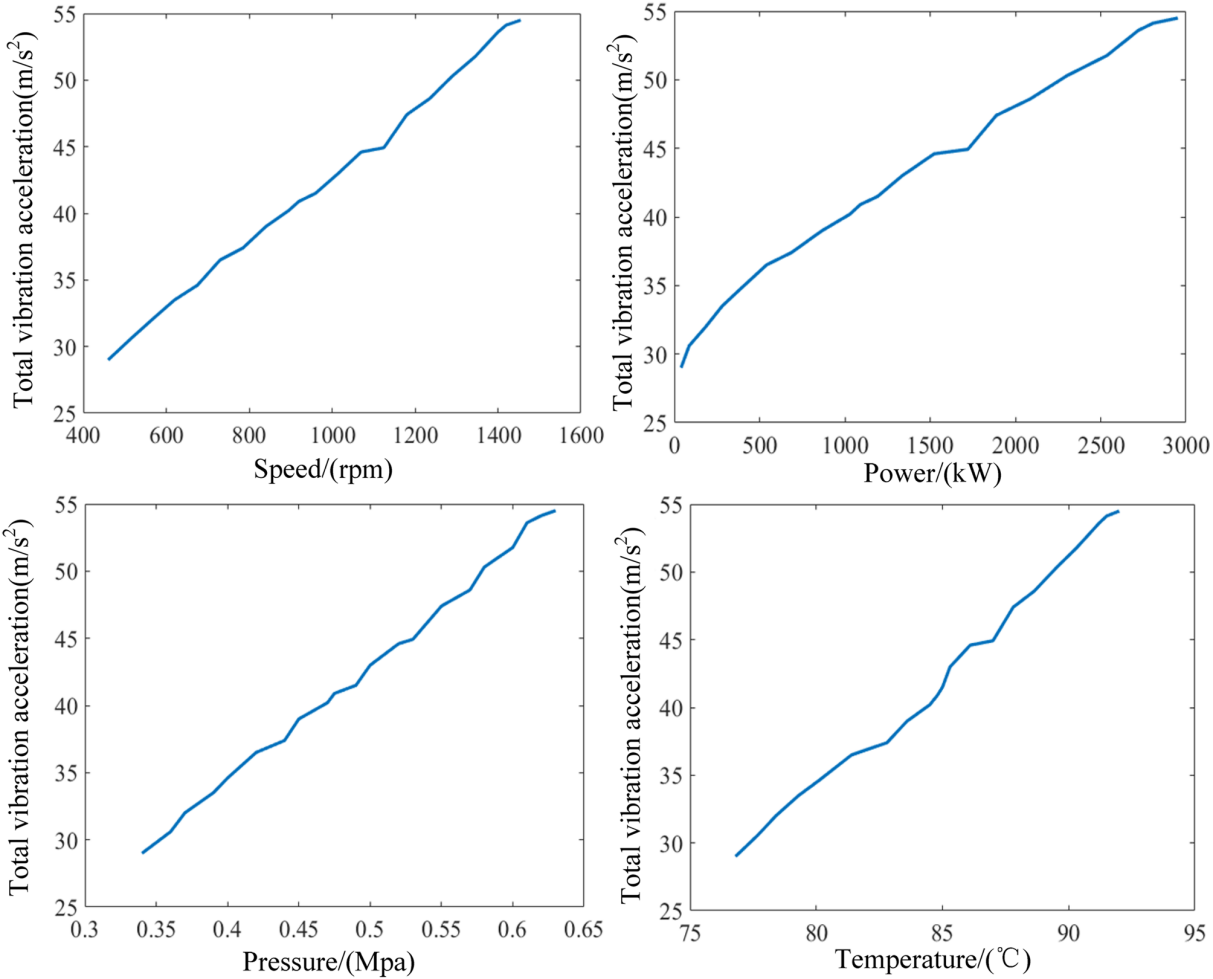
Influence of input parameters on vibration.

Figure 14 shows the effect of all input parameters on the vibration based on the model data. From the Fig. 14, it can be observed that the total vibration is smaller during the starting phase of the diesel engine, with a minimum value of 29 m/s^2^. As the diesel engine runs to the rated condition, the vibration increases to the maximum value of 54.8 m/s^2^. Besides, the output parameters are overall positively correlated with the variation of vibration. This is attributed to the gradual increase in lubricant temperature and pressure as the speed of the diesel engine increases. Moreover, the torque become progressively larger with the rise of the inertia force. This situation makes the piston motion more violent currently, which is the main vibration source of the diesel engine. The trend in vibration was also found in the Ref.^29^.

#### Sound pressure level of the engine

**Figure 15:**
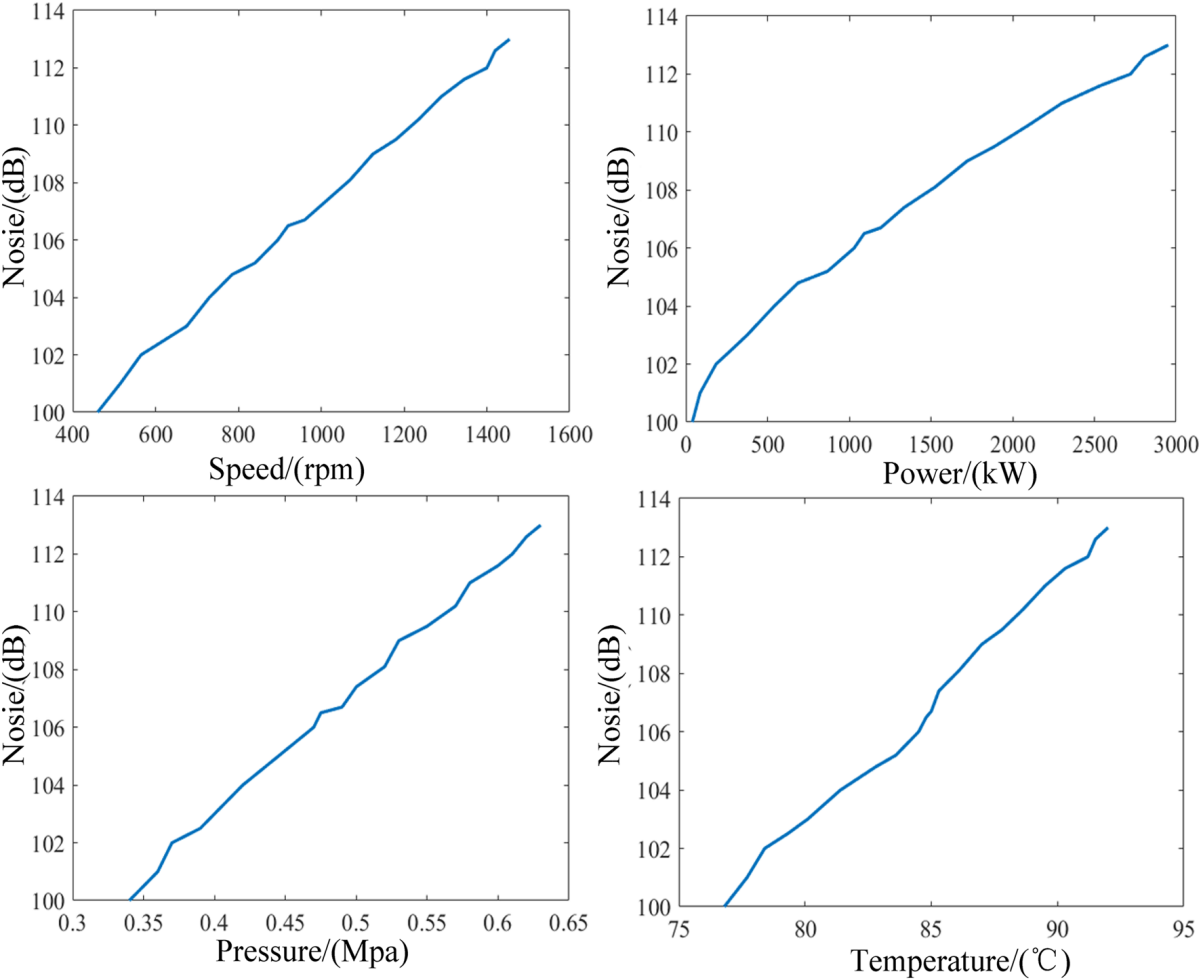
Influence of input parameters on noise.

The variation of noise with the input parameters is shown in Fig. 15. It can be seen from the graph that the sound pressure level of diesel engine rises with increasing speed, power, and lubricating oil temperature. The sound pressure level is basically the same as that of vibration. This trend in brake specific fuel consumption is also validated in Ref.^21^. Meanwhile, it is found that the minimum noise is 100 dB which presents at 460 rpm of speed. The maximum value is 113 dB at the maximum speed. This is attributed to the fact that the decay of sound pressure level may be related to the vibration decay of the diesel engine.

#### Impact factors analysis

Although the parametric effect on the performance of diesel engines has been discussed, the rank of input parameters on performances is still ambiguous. In this paper, the mean influence value (MIV) is used to determine the magnitude of the influence of input neurons on output neurons. The *MIV*_*i*_ can be derived as following^30,31^,

19 $$ MIV_{i} = abs\left( {mean\left( {Ri_{\max } - Ri_{\min } } \right)} \right), $$

where *Ri*_*max*_ and *Ri*_*min*_ are new samples by adding and subtracted by KI on the basis of the original samples. Here KI represents the increment of input parameters.

In this study, four adjustment rates of MIV are set, which include K1 = 5%, K2 = 10%, K3 = 15%, and K4 = 20%, respectively. For each adjustment rate, multiple test trials are conducted to achieve the mean value. Finally, the |MIV| of each variable is calculated. The detailed results are listed in Table 7.

**Table 7:** Value of |MIV| for input parameters.

Output	Input			
	Speed	Power	Lubricating oil temperature	Lubricating oil temperature
Brake specific fuel consumption	0.2225	0.2510	0.1750	0.1895
Total vibration	0.25	0.2	0.1975	0.1972
Noise	0.24	0.188	0.1731	0.1875

**Figure 16:**
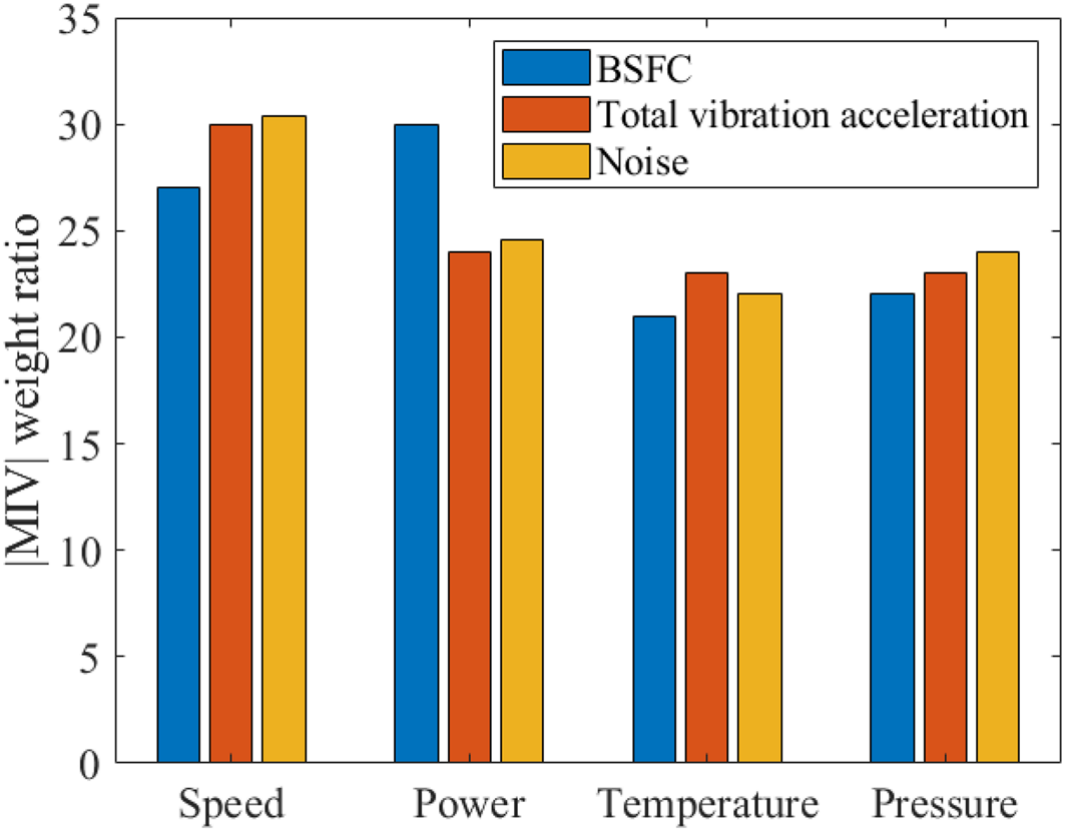
MIV weighting ratio of the input parameters.

Figure 16 shows the |MIV| weight ratio of input parameters on performances of diesel engine. It can be seen from Fig. 16 that the weight ratios of speed on vibration and noise are relatively larger, followed by power. While the weight ratios of lubricating oil temperature and pressure on vibration and noise are relatively small. As for the brake specific fuel consumption, the power takes on a slightly significant weighting, followed by the speed. While the parameters that have less influence on brake specific fuel consumption are the lubricating oil pressure and temperature. Besides, the |MIV| standard deviation of the four input variables is high that indicates the performance indexes are more sensitive to all of input parameters. This also illustrates that brake specific fuel consumption, vibration, and noise are neither affected by a single factor, nor by the combination of multi-parameter.

## Conclusions and future directions

In this study, a hybrid approach combining DNN and VSG is used to achieve an accurate performance model for marine diesel engines. The effects of diesel engine parameters on performances are analyzed, which could provide a guide for engineers in performance assessment and fault diagnosis. The main conclusions of this study can be summarized as follows.

1. The sample data diffused by the VSG method keeps a high accuracy due to an error less than 7%. This indicates that the VSG method is capable of better diffusion of the experimental data. Further, the performance models of marine diesel engine are established based on the proposed hybrid method of DNN coupled with VSG. The coefficients of determination (R^2^) between the predicted and experimental values of fuel consumption rate, vibration, and noise are 0.90, 0.94 and 0.91, respectively. The overall prediction accuracy is more than 93%. The results indicate the proposed model can be effectively applied for predicting and assessing performances of marine diesel engine.
2. Based on the DNN models of performances, the effect of diesel engine parameters on performance is discussed. With the increase of speed, power, lubricating oil temperature and pressure, the fuel consumption rate reduces moderately. Moreover, the change of vibration is in general positively related to the diesel engine parameters. It is also found that the noise attenuation trend is parallel to the changing trend of cylinder head vibration.
3. The MIV algorithm has been used to quantify the weighting of the influence of each input parameter on the diesel engine performance parameters. The results show that speed has the greatest effect on vibration and noise with a weighting of 30% and 30.5% of the four input factors, respectively. While brake specific fuel consumption is sensitive to power due to a weighting of 30%. Moreover, the MIV standard deviation indicates the all of performance indexes are more sensitive to the input parameters.

This paper achieves the prediction of diesel engine performance and emissions, which effectively reduces time consumption and test costs. However, due to the limited experimental budget, only four input variables are designed in this paper, and the effects of multiple input parameters such as compression ratio, combustion starting point, and injection timing, are not considered. Moreover, due to the application of VSG technology, the four input variables selected in this paper are coupled each other, which makes it difficult to carry out the optimal design. However, the method proposed in this paper can be applied for optimizing, design, and analysis of independent input variables as well. Therefore, the research in this paper provides a foundation for the next work. Firstly, the method proposed in this paper provides a modeling basis for the optimization of complex diesel engine system parameters in the future work. Secondly, the results of this paper can provide a basis for performance evaluation and fault diagnosis of the subsequent diesel engine in the next work.
